# Pelvic floor muscle training for female urinary incontinence: development of a programme theory from a longitudinal qualitative case study

**DOI:** 10.1186/s12905-024-03308-4

**Published:** 2024-08-31

**Authors:** Carol Bugge, Jean Hay-Smith, Suzanne Hagen, Aileen Grant, Anne Taylor, Sarah Dean

**Affiliations:** 1 Department of Nursing and Community Health, School of Health and Life Sciences, Glasgow Caledonian University, Cowcaddens Road, Glasgow, G4 0BA UK; 2 Department of Medicine, University of Otago Wellington, Wellington, 6242 New Zealand; 3 Nursing, Midwifery and Allied Health Professions Research Unit, Glasgow Caledonian University, Cowcaddens Road, Glasgow, G4 0BA UK; 4 School of Nursing, Midwifery and Paramedic Practice, Robert Gordon University, Garthdee Road, Aberdeen, AB10 7QB UK; 5 Faculty of Health Sciences and Sport, University of Stirling, Stirling, FK9 4LA UK; 6 Department of Health and Community Sciences, University of Exeter Medical School, St Lukes Campus, Exeter, EX1 2LU UK

**Keywords:** Urinary incontinence, Self-efficacy, Adherence, Process evaluation, Programme theory, Case study, Qualitative, United Kingdom

## Abstract

**Background:**

Urinary incontinence (UI) negatively affects the well-being of women globally. Pelvic Floor Muscle Training (PFMT) is a complex intervention that aims to decrease UI symptoms. Information about how the multiple complex components involved in PFMT achieve and maintain the desired effect are rarely studied as a whole. The evidence base lacks data about how women experience PFMT over time and in the longer-term. This study explored women’s experiences of biofeedback-assisted PFMT and PFMT alone, to identify and understand what influenced self-reported adherence to PFMT, and UI outcomes over time.

**Methods:**

This rigorous longitudinal qualitative case study, nested within a randomised controlled trial, recruited forty cases (women with stress or mixed UI; 20 in biofeedback-assisted and 20 in PFMT alone group). A case included up to four semi-structured interviews with each woman (prior to starting PFMT, end of treatment [6 months], 12 months, 24 months). Analysis followed case study analytic traditions, resulting in a Programme Theory about PFMT from the perspectives of women with UI.

**Findings:**

The theory demonstrates factors that motivated women to seek UI treatment, and how these influenced long-term adherence. Therapists who delivered PFMT played a crucial role in supporting women to know how to undertake PFMT (to have capability). Some, but not all, women developed self-efficacy for PFMT. Where women did not have PFMT self-efficacy, adherence tended to be poor. When women had PFMT self-efficacy, the conditions to support adherence were present, but contextual factors could still intercede to inhibit adherence. The intercession of contextual factors was individual to a woman and her life, meaning any particular contextual factor had inconsistent influences on PFMT adherence over time for individual women and exerted varying influences across different women.

**Conclusion:**

Long term adherence to PFMT is a complex interaction between many different factors. Enquiring about an individual woman’s motivation to seek treatment and understanding the contextual factors that affect an individual woman will enable a practitioner to support longer-term adherence.

**Supplementary Information:**

The online version contains supplementary material available at 10.1186/s12905-024-03308-4.

## Introduction

Urinary incontinence (UI) is a common, global health issue for women with prevalence estimates ranging from 5–69% [1]. UI negatively affects women’s quality of life across different countries and sociodemographic characteristics [1–4]. UI treatments include surgery, medication, conservative therapies—commonly pelvic floor muscle training (PFMT)—and lifestyle interventions [1]. If there is adherence, PFMT is an effective UI treatment in the short-term [1, 5].

Adherence is defined as the degree to which a person’s behaviour “corresponds with the agreed recommendations from a healthcare provider” [6]. For PFMT to be effective at strengthening muscles, adherence over time is required to achieve and sustain the required muscle changes [7–9]. Three reviews of qualitative evidence have aimed to: identify barriers and facilitators to PFMT in adults [8]; understand how women with pelvic floor dysfunction experience conservative treatments [7]; and to synthesise findings exploring the experience of, and adherence to, PFMT [10]. None of these reviews focussed specifically on supervised PFMT for women with UI, but all included such studies, and each identified factors that influence PFMT adherence. The factors, although conceptualised differently in each review, include notions of: capability to undertake PFMT e.g. knowledge, understanding and skills to do PFMT; opportunity to undertake PFMT e.g. external factors that may support or inhibit a woman’s ability to do PFMT regularly; and motivation e.g. linked to the way PFMT is delivered [7, 8, 10]. The quality of the included studies across the reviews was noted to be limited [7]; moderate to high [8]; and variable [10]. Evidence from other studies that focus on adherence suggests patient-related factors are important in PFMT adherence e.g. motivation and household income [11–13].

Within the reviews, there was limited evidence that enabled exploration of adherence, and the contextual factors that influence it, over time and in the longer-term. Most studies included one-off interviews that were at, or near to, completion of the supervised PFMT treatment period. One recent qualitative study (*n* = 6 participants, pre and post treatment interviews) [14] and one quantitative study (*n* = 647 continent women over 55 years, assessed at 3, 12, and 24 months) [13] offer longitudinal data. A small number of studies consider longer term views after PFMT treatment: for example, a study of 12 participants, 2 years post treatment [15], a cross sectional study of 61 participants who received PFMT 6 months to 2 years earlier [16] and a study of 31 participants, 6 months post-treatment [17]. These studies identified the influence of contextual factors, such as competing demands from work or home, on longer-term adherence.

Self-efficacy is argued to be important for managing UI [18] and for PFMT adherence [7, 8, 19]. Self-efficacy is an individual’s belief in their ability to undertake behaviours required to achieve the desired outcome [20]; in this case PFMT behaviours to lessen/cure UI. There are four documented self-efficacy sources: performance accomplishments, vicarious experience, verbal persuasion, physiological states [20]. PFMT self-efficacy may support more PFMT behaviour (i.e. adherence), thus these are important components in a pathway linking PFMT delivery to treatment outcome [8]. As adherence over time is central to treatment effect, and self-efficacy and context are important to adherence, it is important to develop a greater understanding of women’s experiences of self-efficacy, context and adherence to PFMT over time and in the longer-term in a way that enables consideration of the complex interacting factors.

The UK Medical Research Council guidance for developing and evaluating complex interventions highlights the importance of developing a Programme Theory that explains the interaction between a complex intervention, the wider context and the desired outcomes [21, 22]. While there is some evidence that permits the development of an initial Programme Theory, longitudinal data are needed to support understanding of how the links between PFMT self-efficacy, adherence, context and UI outcomes unfold in the longer-term.

**Fig. 1:**
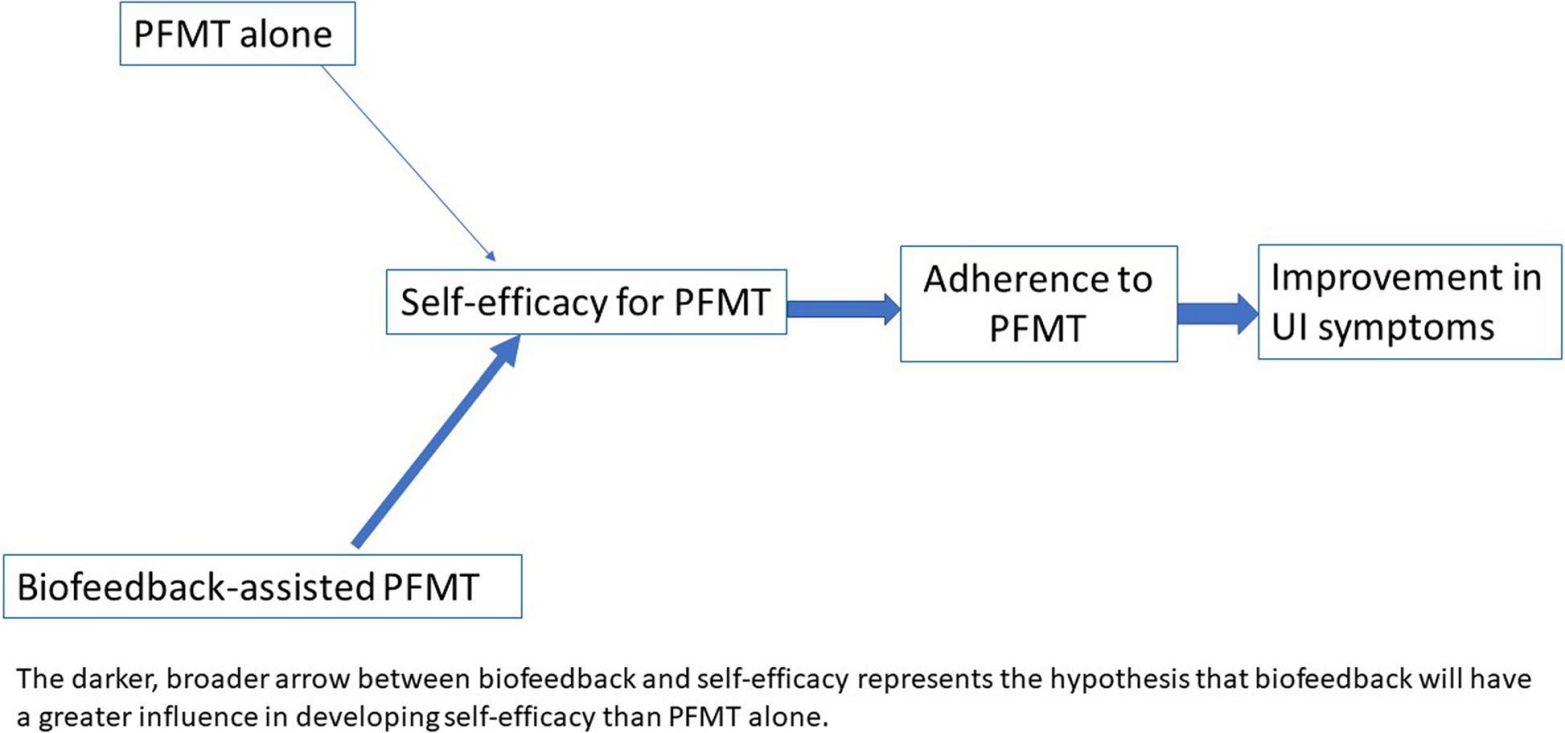
Proposed mechanism of action showing hypothesised pathway from intervention to outcome underpinned by Self-Efficacy Theory

The OPAL randomised controlled trial (RCT) evaluated the effectiveness of biofeedback-assisted PFMT (used in clinic and at home) compared to PFMT alone [23]. For biofeedback, a vaginal probe sensed the pelvic floor contraction and provided visual information to the woman about pelvic floor muscle contraction performance. All women received therapist feedback on pelvic floor muscle contraction from vaginal palpation, and were offered six appointments with a therapist delivering PFMT over 16 weeks. The intervention development drew upon the Information Motivation Behaviour Framework [24] and Behaviour Change Techniques [25] and aimed to enhance women’s self-efficacy and adherence (see Fig. 1). The primary trial outcome was continence severity at 24 months post-randomisation. Secondary outcomes included PFMT self-efficacy. A longitudinal qualitative case study ran concurrently with the trial.

In this paper, analysis of case study data provides evidence regarding women’s experiences of PFMT over time, resulting in a Programme Theory connecting PFMT self-efficacy, adherence, context and UI outcome for two years from the outset of treatment.

## Methods

### Study aim

The study investigated women’s experiences of the OPAL trial interventions, PFMT alone and biofeedback-assisted PFMT, to: identify barriers and facilitators to adherence in the short- and long-term; explain the process through which they influenced adherence; and identify whether they differed between randomised groups.

### Study design

A longitudinal, qualitative multiple case study was conducted [26, 27] in parallel to the RCT, which also had a mixed method process evaluation [28, 29]. Case study design followed the traditions reported by Yin [27] and was advocated because the question aimed to understand the detail of *how* and *why* barriers/facilitators influenced adherence. Yin [27] advocates the use of theoretical propositions in analysis which further facilitated links to a hypothesis driven RCT analysis. The longitudinal design, mirrored the trial, and was used to give an understanding of influences evolving over time, in-depth for an individual woman [30]. Thus, the longitudinal case study design supported an in-depth exploration and analysis surrounding PFMT, a complex intervention, in the detailed context of a woman’s life to understand the processes through which women developed self-efficacy and / or adherence and how this evolved over time [27, 31, 32].

The study was conceived and funded prior to the current iteration of the Complex Intervention Framework [21]. Drawing upon the earlier process evaluation guidance [22, 33], a mechanism of action was hypothesised (Fig. 1) as follows. Biofeedback-assisted PFMT would improve self-efficacy for PFMT contraction and training more than PFMT alone. Biofeedback is a named behaviour change technique [25] that provides physiological information (sensory, visual and auditory) about the contraction in muscles which are otherwise ‘hidden’. The protocol for the clinician’s use of biofeedback offered further opportunity for more behavioural change support (e.g. feedback on behaviour and outcomes by summarising information downloaded from home biofeedback machine use). Therefore, biofeedback and the way it was used, (i.e. women get feedback on how doing PFMT affects their physiological muscle state) would act as a performance accomplishment source of PFMT self-efficacy. These proposed increases in PFMT self-efficacy would lead to more PFMT behaviour and long-term adherence, which in turn would lead to greater improvement in UI symptoms.

Ethical approval was granted on 13.03.13 (13/WS/0048). No additional ethical concerns arose during the study.

### Cases

Eligibility criteria for women in the trial are presented in Table 1. In the trial, 600 women were randomised, 300 in each group from 23 centres across the UK.

**Table 1:** Eligibility criteria for the trial

Inclusion criteria	Exclusion criteria
• Women • Aged 18 years or older • Woman presents with a new episode of stress UI (SUI) or mixed UI (MUI)	• Symptoms of urgency UI alone • Woman has received formal PFMT instruction in the previous year (e.g. from a physiotherapist) • Woman is unable to contract her pelvic floor muscles • Pregnant or < 6 months post-natal • Pelvic organ prolapse greater than stage II on POP-Q measurement • Receiving active treatment for pelvic cancer • Cognitive impairment that affects a woman’s ability to give informed consent • Neurological disease (Multiple Sclerosis, Parkinson’s Disease, Stroke, Motor Neurone Disease, Spinal Cord Injury) • Known nickel allergy or sensitivity • Already participating in other UI research.

The case study purposively sampled from within the trial participant group; hence, all inclusion and exclusion criteria for the trial also apply to the women within the case study. Purposive sampling used maximum variation based on: randomised group [representation from biofeedback-assisted PFMT and PFMT alone groups]; treatment centre type [representation from women treated in large urban University Hospitals, more local District General Hospitals and local Community Clinics]; UI type [women with SUI and MUI], and therapist type [women treated by Physiotherapists and Nurses]. We aimed to recruit 40 cases (20 per group) to enable a full and detailed multiple case analysis [27]. An individual case was one woman who was randomised within the trial and included her data across time. Women who indicated interest in the case study (checked box on the trial consent) were sent a letter and Participant Information Leaflet about the case study. Women were contacted by a researcher to answer any questions, and if willing to participate, a suitable date for the first interview was agreed. Written informed consent from each participant was obtained prior to interview and checked prior to each subsequent interview.

#### Data collection

Women were asked to participate in four semi-structured interviews across two years. Women were interviewed at baseline (randomised but not yet attended their first appointment), 6, 12 and 24 months after randomisation, which mirrored the timing of trial outcome measurement. Interviews were semi-structured, digitally recorded, transcribed and undertaken by two experienced researchers: AG (a medical sociologist) and AT (a nurse researcher) who had no previous relationship with the study participants. Where possible, baseline and six-month interviews were face-to-face, either at the participant’s home or in the clinic, and 12- and 24-month interviews were by telephone. Where there was insufficient time to arrange the baseline interview in person prior to the initial appointment, it was undertaken by phone. Interview schedules were developed based on the underpinning theory, the hypothesised mechanism of action and the theoretical proposition that factors influencing adherence would change over time (interview schedules are attached as a Supplementary File). For example, interviews explored women’s experiences of: the social contexts within which they experienced UI; the intervention they received, their belief in their abilities to undertake PFMT, their adherence and outcomes. In all cases interviews explored change over time and reasons for those changes. The semi-structured interview format ensured the key areas of interest were covered at each time-point while interviewers also encouraged women to identify and expand on areas the women identified as important and relevant to them.

#### Data analysis

Data analysis (led by CB, a Professor of Nursing) followed case study analytic traditions [27]. The work presented here, by the current authors, cross references to analyses conducted during the funded period by other members of the research team, which was published in the report to funders [34].

Analysis began with a detailed interrogation of a sub-sample of cases with complete data (i.e. interviews at all time-points; indicated by ^a^ in Table 2). First, the four interviews per case were read and a case summary written into a Microsoft Excel® Workbook by the lead author to capture key areas: overarching context, links between self-efficacy and how therapists delivered care, other influential factors related to self-efficacy, adherence and changing adherence over time, facilitators and barriers to adherence, UI symptoms at outset and over time, other outcomes and change over time, and additional analyst notes. Second, the case summary was re-read, and iterative codes developed within Microsoft Excel® and by creating tables in Microsoft Word®. Third, based on analysis of eight complete cases, further codes were developed, by comparing across cases (CB), that explained the process through which women moved from seeking treatment to their outcome i.e. the initial Programme Theory [21]. Fourth, the initial Programme Theory and codes were documented and discussed between three authors (CB, SD, JHS); in this way disagreements on meaning of codes were resolved through discussion. Fifth, the theory was further developed based on the analysis of six more complete cases (by CB). Codes were documented in tables and were adapted and expanded as analysis progressed. Sixth, two further cases were analysed to test the theory. That theory was subject to further discussion (between four authors: CB, SD, JHS, SH), which included theoretical and clinical insights. This detailed analysis included review of 16 complete cases. The additional 24 cases were then reviewed by the lead author to address three questions:

1. Is there anything in this case that challenges the developed theory?
2. In what ways, if any, does this case add understanding within the developed components of the theory e.g. are there other contextual influences on women’s PFMT self-efficacy?
3. Does the case offer stronger examples of the theory, or codes within it, that would better represent the data for publication?

**Table 2:** Summary of cases

**Case ID**	**Trial group** *Biofeedback: Biofeedback assisted PFMT* *PFMT: PFMT alone*	**Type of UI** *SUI: Stress UI* *MUI: Mixed UI*	**Age** *(at baseline)*	**Number of completed interviews** *A: pre-treatment* *B: 6-month (immediate) post treatment* *C: 12 months* *D: 24 months*
1^a^	Biofeedback	SUI	65	4: A,B,C,D
2^a^	Biofeedback	SUI	39	4: A,B,C,D
3^a^	PFMT	SUI	40	4: A,B,C,D
4^a^	PFMT	MUI	57	4: A,B,C,D
5	Biofeedback	SUI	57	3: A,C,D
6^a^	PFMT	SUI	76	4: A,B,C,D
7	Biofeedback	MUI	44	3: A,B,C
8	Biofeedback	MUI	49	4: A,B,C,D
9	PFMT	MUI	52	1: A
10	PFMT	MUI	43	4: A,B,C,D
11	PFMT	MUI	20	3: A,B,C
12	PFMT	MUI	66	1: A
13^a^	Biofeedback	SUI	55	4: A,B,C,D
14	PFMT	SUI	51	4: A,B,C,D
15	PFMT	MUI	52	4: A,B,C,D
16	Biofeedback	MUI	25	2: A,B
17^a^	Biofeedback	MUI	56	4: A,B,C,D
18	Biofeedback	MUI	31	3: A,B,C
19	PFMT	SUI	35	4: A,B,C,D
20^a^	PFMT	MUI	65	4: A,B,C,D
21	Biofeedback	MUI	43	2: A,B
22	PFMT	MUI	40	1: A
23^a^	Biofeedback	MUI	45	4: A,B,C,D
24^a^	PFMT	MUI	42	4: A,B,C,D
25^a^	PFMT	SUI	42	4: A,B,C,D
26	PFMT	MUI	57	4: A,B,C,D
27^a^	Biofeedback	MUI	28	4: A,B,C,D
28^a^	Biofeedback	MUI	36	4: A,B,C,D
29	Biofeedback	SUI	53	1: A
30	Biofeedback	MUI	39	2: A,B
31	Biofeedback	MUI	37	1: A
32	Biofeedback	MUI	66	4: A,B,C,D
33	PFMT	MUI	44	2: A,B
34^a^	PFMT	MUI	38	4: A,B,C,D
35	Biofeedback	MUI	69	2: A,B
36^a^	PFMT	MUI	74	4: A,B,C,D
37	Biofeedback	MUI	33	1: A
38	PFMT	MUI	64	4: A,B,C,D
39^a^	Biofeedback	MUI	54	4: A,B,C,D
40	PFMT	SUI	38	1: A

^a^The 16 of 24 complete cases used to develop the Programme Theory

Accordingly, all available data were analysed. Reviewing the additional 24 cases confirmed that the theory remained robust and no overarching elements were added. Some, rare, new understandings within components of the theory were identified and they are included herein. No differences in theoretical constructs between those who participated in all four interviews and those who did not were apparent. Therefore, we are confident that the theory is robust and comprehensive.

Reflexivity was supported by frequent discussions about the evolving analysis, feedback on this manuscript and a journal kept by the main analyst. Rigour was defined using four characteristics of trustworthiness [35]:

- Credibility through prolonged engagement with the data, using all data in the analysis and discussion with peers;
- Dependability and confirmability through using a reflexive journal and discussion of process and findings in meetings with peers; and,
- Transferability by providing a detailed account of the setting and participants.

## Findings

From the trial data analysis there was no evidence of statistical superiority of biofeedback-assisted PFMT compared to PFMT alone for the primary outcome (UI severity at 2 years), although for one secondary outcome (PFMT self-efficacy at two years) there was a difference that favoured biofeedback-assisted PFMT [23]. Case study analysis did compare cases in the biofeedback-assisted PFMT group with those in the PFMT alone group. There were more similarities in factors that influenced the Programme Theory, and its component parts, than there were differences between the groups. Therefore, the data presented below focus on the combined case study dataset, including participants from both trial groups, and draws upon specific evidence related to biofeedback-assisted PFMT or PFMT alone as and when it is relevant to understanding.

### Recruited cases

Forty cases were recruited as planned (20 per trial group) (Table 2). Women were 20–76 years at baseline; 11 had SUI and 29 MUI. Six were treated in community clinics, 16 in University Hospitals and 18 in District General Hospitals. Most women (*n* = 36) were treated by physiotherapists and the remainder by nurses. Twenty-five women completed all four interviews. Due to a technical difficulty with the audio-recorder, full datasets were available for 24 (10 biofeedback-assisted PFMT, 14 PFMT alone). The total dataset contained 125 interviews. Interview recordings per case were 15-126 minutes long. 

### Logic model for the programme theory

**Fig. 2:**
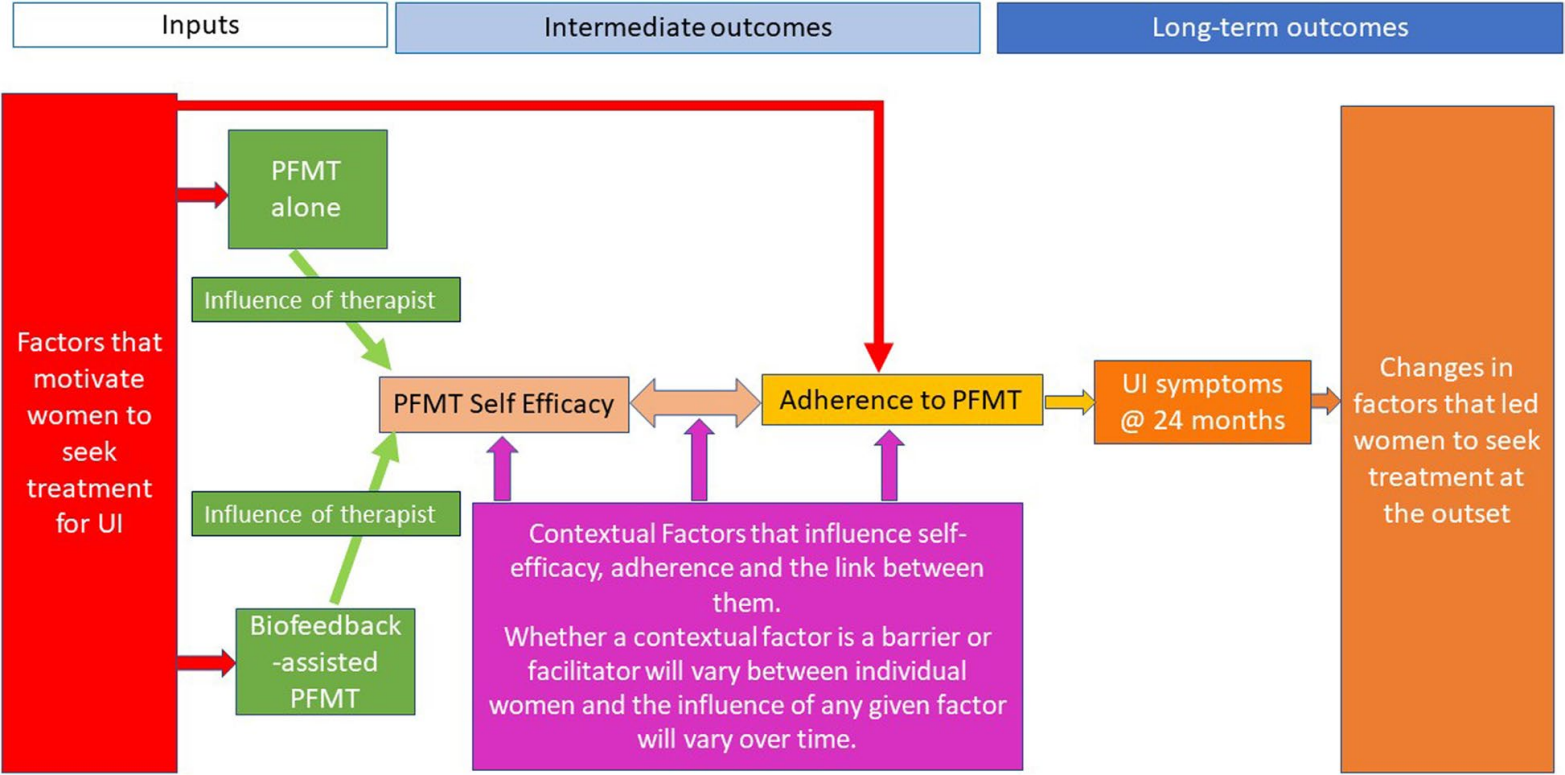
Logic model of the Programme Theory of biofeedback-assisted PFMT and PFMT alone

The logic model for the Programme Theory is presented in Fig. 2 and explained below. Explanation of the components of the model follows.

The model demonstrates that there are inputs for women which start prior to treatment. These inputs are the motivating factors that take women to treatment (in this case trial randomisation). These motivating factors may underpin what is later expressed as their intentions to adhere, or behavioural adherence to prescribed treatment. Women received either biofeedback-assisted PFMT or PFMT alone, delivered by a therapist, and then showed individual variance in appointment attendance. The therapist was central to the development of a woman’s belief in her ability to undertake PFMT and its ability to reduce UI symptoms (i.e. to women gaining PFMT self-efficacy). Analysis identified that all four theorised self-efficacy sources (performance accomplishments, vicarious experience, verbal persuasion, physiological states) were present in the way women developed PFMT self-efficacy [20]. PFMT self-efficacy and adherence were intermediate outcomes on the pathway to the long-term UI outcomes. Contextual factors influenced how women developed self-efficacy or not, the pathway through which self-efficacy and adherence interacted, and adherence itself. The influence of any given contextual factor was not consistent across women, and the influence could alter over time. Based on factors that motivated women to attend treatment, the intervention they received from the therapist, the woman’s self-efficacy and the influencing contextual factors, women then adhered to a greater or lesser extent to PFMT. Some women demonstrated agency in that they adhered as much as they wished to, to achieve their personal goals. This may, or may not, have matched exactly the PFMT prescription by the therapist. Many women believed that PFMT adherence would improve UI outcomes. That is not to say that all women adhered or had improved outcomes, but that they articulated the link between adherence and outcome, and this was true whether they adhered or not. UI outcomes in turn influenced the extent to which the factors that led women to seek treatment were addressed. The case in Table 3 shows the inter-linking of these complex components.

**Table 3:** A case example of the Programme Theory

*Case 27 was a 28-year-old woman who lived with her partner and two children. She sought treatment because she was ‘wetting the bed’. She was motivated to attend treatment because she wanted to resolve UI, be able to get back to the gym and avoid surgery. She cried in the GP practice when telling her GP that she ‘wet the bed’ and her symptoms got her down. She was randomised to biofeedback-assisted PFMT. Case 27 felt that she accomplished performance of the PFMT technique by being taught how to do PFMT properly, and by receiving confirmation she was doing PFMT properly *via* vaginal examination by the therapist. She did not feel the biofeedback helped her achieve performance accomplishment of the pelvic floor muscle contraction, the therapist feedback did that. She found the therapist supportive, someone she could talk to and she felt accountable to her. Case 27 had confidence in her ability to do PFMT correctly right up to 24 months.*
*I feel like if it [UI] got bad again … I've got these exercises to fall back on (Interview D).*
*Case 27 stopped using biofeedback as she didn’t like it. She did adhere to PFMT throughout the supervised period and continued to do PFMT roughly twice a week up to 24 months. Her desire to be dry when physically active was a strong motivator for her from the outset and over time. The key facilitators to adherence were: symptomatic improvement (she could feel improvement in UI symptoms so wanted to continue PFMT to maintain that improvement); having cues (brushing her teeth and going to bed – at 24 months going to bed remained a cue); and her partner knowing she had UI and was learning PFMT. Case 27 found UI and its treatment an embarrassment but when she realised UI was common she felt it became OK. Her busy life and ‘forgetfulness’ were barriers to PFMT adherence. Case 27 improved throughout the study and at 24 months described her UI symptoms as "Pretty much gone, I don’t really suffer anything anymore". From 12 months she said:*
*"I've managed to start going back tae [to] the gym, I starting a yoga class on Friday, … a couple o' [of] months ago I maybe wouldn’t have been confident enough to go " (Interview C)*
*At 24 months she was back to running with no UI symptoms. At 24 months she just didn’t worry about UI as much as she did:*
*" in my head I've, I've made a link that well, I'm doing these exercises so it shouldn’t happen anymore [it's taken a bit of] the panic away … before I used tae [to say] “oh no, am I going to leak, am I going to, what's going to happen?,” so it's, it's been like a safety net I would say" (Interview D).*

### Explanation of the components within the theory and how they interacted

#### Factors that motivate women to seek treatment for UI

While there was recognition within the OPAL intervention that women starting treatment had existing beliefs and understandings (therapists were asked to elicit and address misunderstandings about UI and its treatment), the original mechanism of action started with the allocated treatment to PFMT alone or biofeedback-assisted PFMT (Fig. 1). Data demonstrated that factors motivating treatment-seeking were important drivers in adherence to treatment (i.e. they were motivators that kept women doing PFMT or biofeedback-assisted PFMT over time); for example, if participating in physical activity was an important driver for a woman then it continued to influence her PFMT adherence over time.

The motivating factors related to: UI resolution / prevention of UI getting worse; UI resolution so women could live their lives the way they wanted to; emotional drivers; helping other aspects of health and well-being; and other outside influences.

The women wanted PFMT to resolve UI or prevent it getting worse, avoid or delay surgery, or to use fewer/smaller/less bulky pads. For example:

*I was just fed up with [UI] getting worse … I don’t want to have to stop the exercising, … [I'm going to have to] get some help to try and see if there's anything that could be done … I don’t want to have to wear pads all the time (Case 25 Interview A)*

Women were motivated to do PFMT so that they could live the lives they wanted to lead, for example, so that they could exercise without leaking (Case 3), get up and get on (Case 24), and not leak when having sex (Case 39). Women were motivated by emotional drivers such as feeling less anxiety about UI, reducing the risk of being embarrassed and having confidence to do normal day-to-day activities. Women were hopeful that PFMT might resolve other issues such as back pain (Case 2).

*I din'nae [don’t] really want tae [to] go out until I get everything sorted … or even better than what I am, 'cause I just worry about it and I wouldn’t be able tae [to] enjoy myself (Case 18, interview A)*

Women worldwide contextualise UI as something to be tolerated [3] and women in our sample were no different. Sometimes it took someone else’s suggestion that help was needed to make women seek treatment.

*the second time it happened [leaked when with family] my daughter-in-law … she said to me “you know, .. [you need to get this sorted?],” I said I've already been once a few years ago and the doctor at the time went “oh it's just your age,” you know …. [my daughter in law said] “you need it sorted out, you don’t need to suffer like this,” you know and that's when I went off to the doctor (Case 20 Interview A)*

Each of these factors acted, over time, for individual women to motivate them to continue, but if the motivating factor no longer applied (e.g. they stopped going to an exercise class) then they would stop doing PFMT. This is explained in more detail in the sections below.

### The development of PFMT self-efficacy

Key areas of treatment led to, or detracted from, women’s development of self-efficacy and/or their adherence. Analysis demonstrated that the therapist was a vehicle for change. Other factors were also influential such as the ease of PFMT, women’s sense of accountability and negative aspects of treatment.

#### The therapist as a vehicle for change

The core component of self-efficacy development was the therapist who delivered the treatment. The dataset contained voluminous data of women talking positively about the therapist who delivered PFMT. Table 4 provides illustration about the links between therapist-patient interaction about PFMT and Self-Efficacy Theory. The therapist supported, or detracted from, women developing PFMT self-efficacy. Women saw the therapist as a credible source of information. They described therapists in positive terms such as supportive, non-judgemental, motivational, and as someone who ensured women were ‘at their ease’. Women’s perspectives demonstrated that a therapist’s supportive behaviour enabled a decrease in women’s anxiety about treatment, that in turn may have supported women being in a more optimal physiological and affective state to learn. Women talked about the therapists helping them to find practical ways of fitting PFMT into their day-to-day lives e.g. supporting women to find the cues that would remind them to do PFMT.

*[The therapist] is just tremendous, she's very reassuring, she's very kind, she's a very clear communicator … very, very clear, she doesn't wrap up things in hundreds of words so you get confused … she's very clear about what you need to do. She's very knowledgeable, she explains things so well, I've never met anyone who can explain things so well, …. that was the best thing about it all was seeing [therapist name], knowing that I was doing things right (Case 32 Interview B)*

**Table 4:** Evidence illustrating how Self-Efficacy (SE) Theory was operationalised in therapist-patient interaction

Sources of SE	Examples of how SE can be operationalised in PFMT including use of *Behaviour Change Techniques*	Case data example quote
Performance accomplishment	Therapist provides *Instruction on How to Perform Behaviour*	*[the therapist] explained everything really well, yeah, and she took her time [case 16, Interview A]*
	Therapist offers opportunity to *Practice and Rehearse* technique (muscle contraction, exercise regimen)	*I think the education was amazing, because if you don’t know what is wrong and how to do it you don’t know, you don’t do it … so the education was the principal thing, when you learn how to do and why it's wrong, what is wrong … and then you can do good for your body (Case 13, Interview B)*
	Vaginal palpation with therapist verbal feedback provides *Feedback on Outcome of Behaviour* (correct muscle contraction technique)	*having somebody there, and I think when you're doing exercises and then being able tae [to] feel that it was working, do you know that way when you would get your assessment … and you did have to do them, the exercises, and she could feel the, the difference … I felt … that was good, just to know that you were doing it properly … 'cause you do those exercises and you really don’t know one way or another if you are doing it right (Case 30, interview B)*
	Therapist feedback on exercise provides *Feedback on Behaviour* (completing the exercise dose)	*my physio certainly said that if I'd managed to do the exercises four times’ a day it would probably have made, make more of a difference, but I just found it difficult to do, you know, the proper repetitions four times’ a day, I just found that … too much [case 25, interview B]*
	Biofeedback from vaginal devices provides *Biofeedback* (correct muscle contraction technique, changing muscle performance over time)	*when I was going along to see the therapist … [and she was saying] … “oh, there has been a great improvement and you can see that on the machine that,” that would kinda spur me on a bit to, to sort of do more (case 21, interview B)*
Vicarious experience	Women learn from therapists talking about other cases to emphasise *Salience of consequences* (how other women have managed the condition and its treatment)	*it was unbelievable when I realised how many women do have it … helped me cope a bit better (case 4, interview C)*
Verbal persuasion	Therapist provides *Information about Health Consequences* and acts as a *Credible Source* of information	*She was very knowledgeable, she explained absolutely everything before I even needed to ask any questions [case 3, interview B]*
	Therapist provides *Social Support* (praise)	*to have someone there that is checking the progress and … that you are progressing, it's a great confidence builder [Case 36 Interview B]*
Physiological states	Therapist puts women at ease	*I really liked [the therapist], yeah, she was really, made you comfortable… I wasn't nervous, I wasn't, she made me feel really at ease, which was good, 'cause it's quite a nervous thing and quite embarrassing as well (Case 30, interview B)*
	Vaginal palpation with therapist verbal feedback provides *Feedback on outcome(s) of behaviour* (muscle contraction strength) or Vaginal device connected to computer screen provides *Biofeedback* (muscle contraction strength)	*I suppose just having it and seeing it, you know on you, with the physio, on the screen and hearing it making a noise when you were using it and knowing that you were doing it … or at least it gave you the reassurance you were doing it properly (Case 7, interview B)*

#### What women learned and “knowing” how to do a pelvic floor muscle contraction

Women learned about pelvic anatomy from the therapist and for some this was the most important part of treatment. Women also talked about the importance of learning about other aspects of UI management beyond PFMT, such as fluid management. However, by far the most important aspect of learning for women was how to do PFMT properly (performance accomplishment) and “knowing” that they were doing a pelvic floor muscle contraction properly, and that it was improving, based on therapist feedback (verbal persuasion). It was feedback from the vaginal examination (the physiological state of the contracting muscles) that, in the main, led women to “know” that they were undertaking PFMT in the way that was needed to get treatment effect. For women who had biofeedback-assisted PFMT, feedback from the biofeedback machine had a similar effect (as it too is designed to give feedback on physiological states).

*you've got to try and light up, it's a bit like the fairground thing where you whack the hammer and you've gotta [got to] try and get the light to the top (Case 39 Interview B)*

Although women also talked about the embarrassment of vaginal examination, the benefits of “knowing” outweighed the embarrassment (Case 24). This knowing gave women the belief in their ability to undertake PFMT unsupervised at home (with or without biofeedback).

*It was just having, it was almost like having a personal trainer, the nurse …I think that was the best thing for me; somebody that’s telling you, no, you're doing the exercises right and I can feel an improvement too, I think having, 'cause you can't see, it's not like doing a bicep curl, you can see your biceps getting bigger and getting stronger, you can't really measure it, so having somebody that’s saying naw [no], you're doing fine (Case 34 Interview D)*

#### Vicarious learning

Women also learned the importance of continuing PFMT in the long term from other people, either through reporting from the therapist or directly from others telling them how it had worked for them (vicarious learning experience).

*I'll tell you one thing that was really helpful, is I had a private conversation with a lady … who told me that she's had this problem and she’d religiously followed the exercises and now, you know, several years on [UI] was absolutely no problem for her at all, and I, there's something about having somebody totally independent of, you know, the medics and the, the, the and so on, that, I know it sounds silly but it, it sort of reinforces it in a different kind of way … (Case 14 Interview C)*

#### The ease of PFMT

Women in both trial groups talked about PFMT without biofeedback being easy to do (Case 1). That it could be done anywhere at any time, that suited the woman’s life, facilitated adherence.

*once you got to know how to do the … [PFMT] exercise, … it was easy (Case 36, Interview C)*

Women also talked about accountability to the therapist, which was supported by attending clinic appointments and, for those with biofeedback, by the machine recording PFMT undertaken. Perhaps more powerfully, women felt accountable to themselves, to self-care in ways that promoted their health and well-being (Case 13).

#### Negative views on PFMT

There were considerably more positive reports of treatment than negatives. Negatives impacting women’s self-efficacy and adherence were: not being convinced that PFMT would work from the outset or when treatment effect took longer to occur than anticipated.

*I was a bit sceptical that it [PFMT] would work completely, you know, it would completely cure my symptoms (Case 25 Interview C)*

Not all women were randomised to their preferred treatment, and this influenced belief in, and adherence to, allocated treatment. The trial standardised the number of appointments women received to control for the duration of therapist contact, a possible confounder, but some women thought six appointments was too many while others had wanted more.

Through the above mechanisms within treatment, some women demonstrated PFMT self-efficacy and others did not. Women who articulated a belief in their abilities to undertake PFMT gained performance accomplishment for PFMT. The combination of knowing they had the correct exercise technique and having the underlying knowledge about their training programme seemed to create a dynamic of sustained belief in their ability to perform PFMT correctly that lasted long after formal instruction and the supervised treatment period was over. For example, Case 1 talked through all of her post-treatment interviews about her ability to re-start exercises and knowing that she had the skill to do so. Women embodied knowledge of their strengthened pelvic floor and they linked those changes in their pelvic floor, achieved by adhering to PFMT, to decreased UI symptoms.

*knowing that the exercises help makes me do it more (Case 20 Interview B)*

Even if women believed in their ability to do PFMT this did not necessarily mean that they adhered, some women chose to stop doing PFMT, but most of these women did say they knew what to do. This was evidenced by women restarting PFMT themselves after a break and saying they did not require further clinical appointments because they knew what to do.

*I've maybe had my wee warning call there [experiencing return of UI when stopped doing PFMT], and I think that'll just remind me that … I need tae [to] keep doing them every day (Case 34 Interview C)*

However, those women who did not gain belief in either their PFMT ability or that PFMT would/had worked for them (i.e. they lacked performance accomplishment), tended to do less PFMT. For example, Case 32 felt that her UI was caused by an anatomical structural abnormality. These causal beliefs [36] were more influential and likely countered any opportunity to gain self-efficacy, which is based on the being able to achieve the desired outcome. For this woman, her strong belief that PFMT was not going to help, meant she did not adhere, thereby forgoing any possibility that PFMT would work.

In summary, treatment received by a therapist was linked to the development, or not, of self-efficacy. In turn self-efficacy, or not having self-efficacy for PFMT, was linked to adherence.

### Adherence to PFMT over time

Some women described full adherence to prescribed regimens and others adhered partially, doing what they felt was enough exercise to achieve the effect they desired. This meant the degree of adherence to prescribed regimens over time varied. There were examples of consistent adherence to prescribed regimens over the two-year period. For example, Cases 36 (PFMT alone) and 23 (biofeedback-assisted PFMT) both adhered ‘religiously’ throughout the supervised phase and continued to undertake PFMT at levels above the prescribed maintenance dose throughout the two-year period. However, there were Cases where adherence was poor from the outset or for some Cases PFMT/biofeedback was variable during the supervised phase then ad hoc for the remaining period. Case 25 found it hard to get to appointments, and to do what the therapist asked her to do between appointments. Following treatment, she did PFMT very occasionally. Case 4 adhered throughout the supervised phase, slowly decreased the amount of PFMT she did and then after 12 months her partner had a stroke, and she did not do PFMT at all.

Women demonstrated agency in making decisions over time about how much PFMT to do to manage their UI symptoms such that they could cope and get on with their lives. Women created a balance between the time needed to do PFMT, the symptomatic outcome and the life they wanted to lead. For example, Case 28 followed the prescribed regimen to start with, thereafter she did as much PFMT as she felt was needed to keep her symptoms at bay.

Even when women articulated a link between adherence and outcome, it did not necessarily follow that they adhered. Contextual factors acted as barriers and the knowledge that PFMT would work to reduce symptoms if adherence was maintained was overridden.

*if I was able to do this all the time and really sort o' [of] concentrate on it and have it sort o' [of] planned out during the day, [if I had] time to do the exercises, then I, yeah, I'm sure it would improve, " (Case 15 Interview D)*

In contrast, for other women, this adherence-outcome link was a key motivator to keep them doing PFMT.

*I know that I've got to keep them going otherwise I'm going to slip back, but I know that I can actually fix this again by doing the exercises, so that's useful (Case 5 Interview C)*

When women did not perceive a link between adherence and outcome, then they also could not see the value of doing PFMT, in this situation adherence was ad hoc or non-existent (Case 25).

### Contextual factors that influence self-efficacy, adherence and the link between them over time

Context was important in understanding PFMT within a woman’s life over time. Facilitators for self-efficacy and ongoing adherence were related to: the woman (personal drive, desire to live the life she wants to lead, ability to overcome obstacles); feelings about UI (it’s not only me); having a routine and prompts; and factors within the woman’s lived environment. Barriers could inhibit self-efficacy and/or adherence but they did not necessarily do so. Barriers included: loss of routine or prompts; comorbidity; factors that affect busyness and attention in day-to-day life; negative emotional attitudes to herself, to UI or to treatments; belief that PFMT would not be effective in resolving UI; loss of biofeedback unit at the end of the supervised treatment period and environmental factors.

#### Factors related to the woman

Women were empowered to achieve improvement in their lives. They saw PFMT as something about and for themselves and that they were the only person who could make the change happen.

*I was so determined though, I mean the thing is you've got to want to, to help yourself (Case 20 Interview B)*

The ability to overcome obstacles facilitated short and long-term PFMT adherence. For example, Case 20 maintained a strong sense of self-efficacy and adherence despite experiencing multi-morbidity (including cardiac surgery). For her, the positive outcome she had experienced in her life having resolved UI symptoms continued to fuel PFMT adherence in the longer term. In contrast, although UI could facilitate adherence, it was also a barrier; when women’s UI resolved, they lost that prompt, and for some, that meant PFMT adherence decreased.

*my symptoms have reduced should I say, so I haven't had as much of a, like a physical prompt to remind me to keep doing them (Case 28 Interview C)*

Although multi-morbidity did not necessarily lead to non-adherence; co-existing health issues were sometimes a barrier to self-efficacy and/or adherence. Over the course of two years, women experienced long-term conditions like arthritis or asthma or short-term problems like flu, chest or urinary tract infection. For example, Case 23 had several medical diagnoses, one of which was fibromyalgia. The pain and direct muscle effects of the disease when it flared rendered her unable to do PFMT effectively as did a chest infection where she could not control her breathing. These effects were not consistent over time, rather they fluctuated in their impact on PFMT adherence.

#### Factors related to UI

Realising that UI was a common condition supported adherence. Negative emotional reactions were associated with the hidden nature of UI; women worked hard to keep UI concealed from society in general. Women were at times surprised and often relieved that UI was much more common than they thought pre-treatment. Realising that they were ‘not alone’ acted as a facilitator for undertaking PFMT by altering their mindset about being a person with UI who was taking control and dealing with their UI through PFMT.

*I just learned that I wasn’t alone, that other people had this … (Case 6 Interview B)*

#### Factors related to routines and prompts

Having a PFMT routine was helpful for adherence. Sometimes that routine gave women ‘hooks’ or ‘prompts’ which acted as exercise reminders. Many of these were usual day-to-day activities such as driving to work, brushing one’s teeth or putting the kettle on (Case 17). Others were learned from the trial treatment protocol, e.g. writing PFMT down in a diary. Conversely, loss of cues or routine could limit adherence. The benefits of prompts could be lost if women had a change in routine/or had not established a routine in the first place. Sometimes it was the specific activity associated with urine leakage that prompted adherence, and if this activity (such as running) was no longer undertaken then there was no prompt to adhere. Women could also report losing the motivation to do PFMT when they were content with alternative management (such as containment) that offered a solution and enabled living the lives they wanted to lead (Case 4). Again, these effects varied for individual women across the course of two years.

#### Factors related to the woman’s lived environment

PFMT was facilitated by women having flexibility at home to do PFMT/biofeedback as and when they wanted to. For some this was about having a private space and enough time whereas for others having someone else within their personal environment knowing they were being treated for UI permitted an openness that facilitated adherence. Where women got help from others within their environment, this facilitated adherence e.g. Case 23 waited until her partner came home from work to help her put the biofeedback probe in as she could not do this herself. There were factors in women’s lived environments that acted as barriers. For example, women’s jobs sometimes meant that they did not have easy access to toilets. For others, people in their home could be barriers e.g. a child coming into a room where a woman was trying to undertake PFMT/biofeedback.

The women lived busy lives often juggling work, families, and major life events. Busyness could act to prevent women gaining self-efficacy or adherence. There were multiple examples of this which included Case 1 where she was a partner, mother and she changed her job over the 24-month period. As a result, she stopped undertaking high impact exercise which was her motivator for attending (leakage when exercising) and so she stopped doing PFMT. Women spoke often about having a lack of time to fit PFMT into these busy lives. Many life events occurred for women which fed into their sense of a lack of time. These included changes in the health status of those around them such as unwell parents, partners, children, or stressful life events like moving house, getting divorced and bereavement. The influence of these contextual factors on PFMT adherence could be short lived or could have longer term impact.

*I think life’s just got in the way, you know, things have happened, so, but it's not, I've not treated it as priority myself, which I should do because I know if I don’t do it, I know if I don’t do this in years to come I will need an operation, so that should be enough gumption for me to you know do it (Case 2 Interview C)*

#### Factors related to negative views about how a woman saw herself

Women spoke of themselves at times using negative, self-depreciating terms such as ‘lazy’, having ‘no willpower’, and ‘forgetful’ (Case 13). Women blamed themselves for non-adherence. Women also voiced negative reactions to PFMT and/or biofeedback. For example, one woman was embarrassed because her partner called the biofeedback machine a vibrator (Case 1). Many women were secretive about their UI, often not telling others (Case 39).

The trial treatment phase was 16 weeks. Although many women felt they had received enough treatment, others felt that their performance accomplishment and adherence were supported by clinic visits and the loss of these, and for those with biofeedback the loss of the biofeedback unit (which women only had during active treatment), could act as a barrier.

The evidence above demonstrates that there are many contextual factors that influence a woman’s ability to develop self-efficacy for PFMT, that influence how that self-efficacy interacts with adherence, and also whether and how a woman chooses to adhere to PFMT. The data showed that these factors vary in their influence between women, a contextual factor that may facilitate for one woman, may act as a barrier for another (comorbidity is a good example of this). The factors also vary over time for individual women with some factors having a more consistent influence over time and other factors waxing and waning over a two-year period (such as a health condition that flares and resolves).

### UI symptoms and changes in factors that led to women seeking treatment

Consistent adherence to PFMT over time is needed to achieve and maintain improvements in UI symptoms [7, 8]. Women in the OPAL study discussed outcomes in two ways: they talked about 1) UI outcomes and 2) about improved continence as the vehicle for doing the other things that mattered to them (e.g. Case 27, Table 3). Many women were positive about their UI and other outcomes. Sometimes women’s outcomes were not linked to UI improvement, e.g. Case 4 was delighted she was prescribed pads to contain the UI and although she still leaked urine she could go about her day-to-day life the way she wanted to. For others the outcome was poor, e.g. Case 24 talked about an initial improvement in UI then deterioration, with symptoms at two years worse than when she started; this vexed her as when her symptoms were better she could get up and get on with the day and help other family members.

## Discussion

### Summary of key findings

This study is the first large-scale qualitative study to follow women with UI within a PFMT programme for two years from treatment outset (and 18 months from treatment completion). It confirms that UI is a hidden, stigmatising and life-altering condition for women [2–4, 37]. The study offers a new Programme Theory that brings together the complex interrelated factors of PFMT, self-efficacy, adherence, context and UI outcomes. The factors that motivate women to seek treatment at the outset influence women’s PFMT adherence across time. The therapist is central to development of PFMT self-efficacy that persists in the long-term. Those without PFMT self-efficacy were not likely to adhere in any meaningful way. Where women did have PFMT self-efficacy it enabled, but did not guarantee, adherence. Furthermore, some women who did have belief in their ability to undertake PFMT and achieved a worthwhile outcome did not adhere. The importance of contextual factors in influencing women’s motivation and opportunity to adhere to PFMT over time is established. Contextual factors did not influence adherence in a consistent pattern: there was variability across individuals and time.

### Strengths and limitations

The main strengths of the study are the size and scope of this longitudinal follow-up, with 40 diverse cases providing a large and robust dataset. The theory developed from the analysis of 16 cases was supported by data from the remaining 24 cases, evidencing its robustness. The prospective longitudinal design adds new knowledge about long-term understanding of the ebb and flow of changing contexts for women with UI after supervised PFMT. Women were interviewed at four time-points, up to and including two years from randomisation. This ensured a deep and evolving understanding of their lives and the influence of contexts on PFMT self-efficacy and adherence without retrospective bias. Further, the scope and scale of the study enabled exploration of several important factors simultaneously, specifically PFMT, motivations, self-efficacy, adherence, context and outcomes that enabled their interaction within a programme theory to be developed.

This study sampled participants from within a National Health Service where women attending for treatment were not required to pay. As not all global health systems have the same healthcare model, there may be elements of the findings that differ in other contexts. For example, cost may feature in a developed Programme Theory where services are not free at the point of contact.

The focus of this study was women with UI and did not include clinician or health service management perspectives. Therefore, the study does not offer information into the Programme Theory about health professional or system factors. Finally, the study focussed on women with stress and mixed UI only and therefore the Theory does not articulate factors related to PFMT for urgency UI or other pelvic floor disorders. As such the Theory could be developed if those additional perspectives were sought [21].

### Discussion in relation to the literature

The Programme Theory is a pathway to understanding women’s views and behaviours before, during and in the long-term beyond formal PFMT instruction that offers a new, practical visualisation of how the component parts, at the macro level, interact and provides detailed data to explain those interactions. Recent reviews demonstrate that there are few studies exploring long-term and longitudinal perspectives [7, 8]. Existing studies explore components of the theory (such as women’s experiences of PFMT in the supervised phase [15, 38]), but there is limited evidence that brings the complex component parts together. This Programme Theory as a whole offers a visualisation of the complexity from a longer-term perspective.

While some participants attended PFMT to control UI specifically, for others the motivations to attend were focused on what being UI free meant for their lives more widely. Within the Programme Theory understanding women’s motivation was important because a) it was what women valued and b) the motivators directly influenced long-term adherence. It is recognised that prior knowledge, intention and motivation are influential in adherence generally and PFMT specifically [8, 16, 39–42]. Studies have identified that motivation for PFMT can be a challenge [10, 13]. As women’s initial motivators are a key driving force to seek help, undertake, and importantly sustain, PFMT; understanding and drawing upon these individual motivators is an important consideration for clinical practice.

The Programme Theory places self-efficacy as influential to the success, or failure, of long-term UI outcomes. Self-efficacy is said to be important to PFMT adherence [8, 19] and PFMT adherence to UI outcomes [7, 8, 10]. It was clear from the case study, and other evidence, that if women did not believe that PFMT would work then adherence was limited [11, 14]. Not all women developed PFMT self-efficacy, instead there was a complex relationship between self-efficacy and contextual factors that mediated or moderated women’s adherence to PFMT. Our study supports other evidence that feedback, a recognised Behaviour Change Technique [25], is important to performance accomplishment of the PFMT contraction. ‘Knowing’ how to do a PFMT contraction was important [7, 10]. Healthcare services may see this ‘knowing’ as *the* key focus of care delivery, however in line with the Capability – Opportunity—Motivation-Behaviour framework [8, 39], the case study data demonstrate that having performance accomplishment alone is not enough to ensure adherence, in the short and longer term. This case study supports theory [20] and other PFMT studies [8, 10, 14] that suggest vicarious experiences; management of physiological arousal state and verbal persuasion are also present in the development of PFMT self-efficacy. Thus, PFMT self-efficacy is important in the short and long- term and within the Programme Theory all four components of self-efficacy are important.

In this case study, as elsewhere, adherence was linked to the outcome of PFMT [7, 8]. Current review evidence has a shorter-term focus, however, some more recent studies explored adherence in the longer-term [13, 14, 16, 17]. There are similarities and differences in these study findings to those reported within this paper. For example, these longer-term studies (including our own) demonstrate that adherence is variable across time, with Hay Smith et al. [14] explaining that variance by how women ‘make sense of it all’. Understanding adherence in the longer term necessitates an understanding of context.

Context is known to be important in the implementation of complex interventions in practice [26, 32, 43, 44]. Three good quality reviews have outlined multiple contextual factors that influence adherence [7, 8, 10]. Our data support the findings of these reviews and develops understanding for the longer-term and within the complexity of other important features, such as self-efficacy. While the data from this case study support many of the barriers and facilitators for adherence that are already published, what the depth of the case study reveals is that contextual factors can vary in their effect both for individuals over time and between individuals. What might be a barrier for one woman may be a facilitator for another. There have been trials that aim to develop strategies to support PFMT adherence but they have shown no effect [42, 45, 46]. The detailed case study data suggest that it is not the barrier per se that prevents adherence but rather the woman’s reaction or mindset towards it within the wider context of her life (including gender-based societal expectations [47]), which is constantly changing over time, that influences her perceived opportunity and motivation to adhere. A focus in practice on tailoring to individual women’s context may be important to support long-term adherence, especially for unsupervised phases, on-going maintenance or re-starting PFMT after a break.

### Implications for practice

PFMT includes health behaviours that, in order to have an effect on UI, require women to adhere over time. To adhere, women need to have self-efficacy for PFMT. Moreover, the contextual factors for an individual woman need to enable her self-efficacy and adherence. This is a complex pathway for a complex intervention. Practitioners deliver on parts of this pathway, they support women to have the capability to undertake PFMT; it may be argued that this is the key role for practitioners. However, long-term adherence requires women to have ongoing opportunity and capability to undertake PFMT, and the motivation to continue or restart [39]. To support long-term adherence, practitioners may wish to explore women’s initial motivations for PFMT and work with those factors to support and strengthen women’s motivation, and to explore opportunities within their lives, including strategies or adaptations, to future-proof longer-term adherence and manage any relapse.

## Conclusion

UI is a prevalent, debilitating problem experienced by women worldwide. The Programme Theory developed in this longitudinal case study offers a visual representation of the complexity and connections within a woman’s pathway from her seeking UI treatment to long-term PFMT treatment outcome. Self-efficacy and adherence hold a central position in the pathway to outcome. Contextual factors play a role in influencing an individual woman’s ability to undertake PFMT and understanding those dynamic factors should play a more central role within the formal treatment pathway. This study focussed on the experiences of women with UI; future research should expand the pathway to include health professional and system factors.

### Supplementary Information

Supplementary Material 1.

## Data Availability

As the data in this study are detailed and longitudinal, any shared data would require careful de-identification prior to sharing. To access data, a request should be submitted to the corresponding author (carol.bugge@gcu.ac.uk) with a scientific proposal including objectives. Written proposals will be assessed by members of the study project management group, and by the University Ethics Committee and a decision made about the appropriateness of the request. The data will only be shared after the data sharing agreement is fully executed.

## Abbreviations

MUI: Mixed Urinary Incontinence
PFMT: Pelvic Floor Muscle Training
RCT: Randomised Controlled Trial
SUI: Stress Urinary Incontinence
UI: Urinary Incontinence
