# Supplementary material for: Pelvic floor muscle training for female urinary incontinence: development of a programme theory from a longitudinal qualitative case study
Source: BMC Womens Health. 2024 Aug 31;24:478. doi: 10.1186/s12905-024-03308-4 (PMC11365274; doi:10.1186/s12905-024-03308-4)
Supplement: Supplementary file 1 — Supplementary Material 1. [file 12905_2024_3308_MOESM1_ESM.docx]

**Exploring women’s experiences of symptoms and treatment: An interview study linked to the OPAL trial**

# Pre Treatment Interview (Interview A)

**RESEARCH QUESTION: To investigate women’s experiences of the interventions, both basic and intensive PFMT, to identify the barriers and facilitators which impact on adherence in the short- and long-term, to explain the process through which they influence adherence, and to identify whether these differ between randomised groups.**

## Introduction to study and self

Thank you for agreeing to meet with me. We greatly appreciate your willingness to help with the OPAL interview study. The OPAL interview study is about women’s experience of UI and how they have got on with the treatment they have been given as part of the OPAL intervention study. I am xxx, one of the researchers on the OPAL study.

## Consent

Go over study and what is involved. Do you have any questions for me? Are you still happy to be interviewed and for that interview to be tape recorded? If yes to all – **ask to sign consent**.

## Introduction to interview

Today’s interview is about your experience of UI and what you hope for from the treatment you are about to have. It will take approximately 30 minutes.

## Ice breaker

How is your health generally?

## Woman’s experience of UI & Symptoms

- When did you start experiencing UI?
- Why do you think it started happening/ what do you think is the cause (Perceived causes)
- Extent of UI symptoms now
- Do symptoms bother you? Extent. Where and when most/least bothersome and why. (anything context/ situation specific).
- Progression over time (both what has happened [past tense] and what they think will happen [will it get better, worse])
- External influences:
  - Where get information about UI? (what you seek/ what you get given / sources e.g. web, magazine, other women etc.)
  - Who else knows about your UI?
  - Explore support from others, who/ what support offered?
  - Does your UI affect others close to you (family, friends)?
- What made her seek help?

## Current Self-Care

- What do you do when you leak urine (explore specific example if possible)
- Do you do anything to manage your UI (deal with it /make it better/ to contain it)
- Does anything you do make it worse
- Anything tried in the past
  - Routines (such as going to the toilet before leaving house/ knowing where toilets are)
  - Containment (use pads etc)
  - Medication
  - Exercise (probe for PFMT specifically and sense of extent to which exercise is generally part of their life)
  - Surgery
- Confidence in managing UI (self efficacy)

## Expectations of Treatment

- What do you understand / know about treatment for UI and PFMT in particular
- What do expect the recommended treatment to be? (probe PFMT and for intervention group biofeedback)
- Can you describe what you think the treatment will be like for you? (practical, clinical, feelings)
- What do you hope to get from treatment? (try to identify main outcome wants to change/ why this or these outcome(s) most important to her)
- Expect to happen (processes of health care and do they think/expect improvement or not)
- Want to happen (in this treatment and do they want an operation/medication instead or afterwards)
- Anticipate anything that might influence treatment (e.g. ability to attend, ability to exercise at home)
- what will make it easier for you / what will make it more difficult for you

## Closure

Thank you for talking with me today. The interview we have recorded will be removed from the recording device as soon as possible and stored securely. When the interview is typed up (transcribed), all identifying information will be removed. We will study the information you have given us alongside that given by other women. We would like to speak with you again in 6 months time, in that interview we will talk about how you have got on with the treatment. We can come to your home or to the clinic, whichever you would prefer. I will call you to make that appointment; confirm consent to call.

**Exploring women’s experiences of symptoms and treatment: An interview study linked to the OPAL trial**

# Post Treatment Interview (Interview B)

**RESEARCH QUESTION: To investigate women’s experiences of the interventions, both basic and intensive PFMT, to identify the barriers and facilitators which impact on adherence in the short- and long-term, to explain the process through which they influence adherence, and to identify whether these differ between randomised groups.**

## Re-introduction to self, interview, consent

Thank you for agreeing to see me again. We greatly appreciate the help you are giving with the OPAL study. To recap the OPAL interview study is about women’s experience of urine leakage and how they have got on with the treatment they have been given. I am xxx, one of the researchers on the OPAL study. Today’s interview will take approximately one hour. Are you still happy to be interviewed? Do you have any questions for me before we start?

## Ice breaker

How have you been generally since we last spoke?

## Symptoms

- Extent of UI symptoms now
- Comparison of symptoms now to 6 months ago (ie pre treatment)
- Perceptions of stages of change (ie when noticed, what changed)
- Why do you think things have changed/not changed? (probe for things in relation to:
  - social [e.g. family support];
  - intervention [were there things about the intervention that the person thinks are related to change];
  - confidence to undertake exercise/ manage leakage [self-efficacy];
  - lifestyle/ self-management (e.g. fluid, dietary changes).

## Intervention

- General views on intervention (How did you find the treatment you received?)
- Probe about PFMT – features positive, features not so helpful
  - What was it like being taught the exercises
  - How did you get on learning the exercises? (check confidence in technique) (mastery)
- For intensive – probe about biofeedback – features positive/ features not so helpful
- What most helpful about the treatment? (probe: exercise/ therapist or nurse/ biofeedback if got it/ feedback on progress etc)
- Did you have any concerns about treatment (probes exercise/ therapist or nurse/ biofeedback if got/ feedback on progress etc)
- Anything change about treatment (probes for exercise etc)? why?
- Experience of service delivery context (appointment system, privacy ….)
- Explore perceptions of relationship with therapist
- Anything outside the service delivery that influenced experience of treatment – external influences
- If they did not complete treatment but remained in study why?

## Appointments

- Adherence to appointments (did you manage to attend all the appointments)
  - Opinions on number of appointments (too many/ too few)
  - Opinions of exercise asked to do at home – too much/ too little [including biofeedback]
- Factors that affected ability to attend/ not attend scheduled appointments
  - - Social influences e.g. family commitments
    - Environmental influences eg. ability to travel?
    - Your own confidence [Self efficacy] influences?

## Self Care

- PFMT undertaken at home:
  - Experience of doing exercise at home ( detail – where, when, how often)
  - what was easy? What was difficult?
- If intensive – biofeedback undertaken at home
  - Experience of doing biofeedback as part of exercise regimen at home ( detail – where, when, how often)
  - Explore experience with biofeedback and experience without
  - what was easy? What was difficult?
- Any other ways you manage UI?
- Factors influencing adherence to home programme of exercise
  - things that helped you stick with exercise,
  - things that stopped/hampered exercise
  - Did you manage to form a routine for exercise? What was it? How work for you? [questions about maintenance]
  - Were there breaks in your exercise routine (illness/ holiday)? Explore why there was a break and actions taken to re-start exercises (questions about relapse management)?
  - Was there anyone to help you stick to your exercise programme? Or did anyone hinder your ability to do the exercises?
  - Other social influences (such as work commitments etc)
  - Other environmental influences (such as privacy at home etc)
  - How is your confidence to exercise now? Has it changed over time?
  - Do you plan to continue exercising? Explore what will do? How will do?

## Links between intervention and outcome (if not been explicitly covered through previous content)

- Links between intervention and outcome: what are they perceived to be; how do they make a difference; why do they make a difference?
- What was it like to take part in the research study (more generally)

## Closure

Thank you for talking with me today. The interview we have recorded will be removed from the recording device as soon as possible and stored securely. When the interview is typed up, all identifying information will be removed. We will study the information you have given us alongside that given by other women. We would like to speak with you again in 6 months time, that interview is usually undertaken by phone (explore best times to call/ make an appointment to do); confirm consent to call.

**Exploring women’s experiences of symptoms and treatment: An interview study linked to the OPAL trial**

# 12 month Follow-up Interview (Interview C)

**RESEARCH QUESTION: To investigate women’s experiences of the interventions, both basic and intensive PFMT, to identify the barriers and facilitators which impact on adherence in the short- and long-term, to explain the process through which they influence adherence, and to identify whether these differ between randomised groups.**

## Re-introduction to self, interview, consent

Is this an OK time to call? Is there another time I can call back?

Thank you for agreeing to talk with me again. We greatly appreciate the help you are giving with the OPAL study. To recap the OPAL interview study is about women’s experience of UI and how they have got on with the treatment they have been given. I am xxx, one of the researchers on the OPAL study. Today’s interview will take approximately 15 minutes – is it still OK to record? Are you still happy to be interviewed? Do you have any questions for me before we start?

**In this interview we will focus on the last six months (that is since we last spoke to you).**

## Ice breaker

How have you been generally since we last spoke?

## Symptoms [maintain focus on comparison to 6 months ago]

- Extent of UI symptoms now/ how bothered by them
- Comparison of symptoms now to 6 months ago (ie post intervention)
- Why do you think symptoms have changed/not changed over the last 6 months? [Focus on issues woman raised at 6 month interview and ask about intervention].

## Intervention

- Thinking back to treatment with therapist/nurse, what are your views on it now.
  - - PFMT (positive features, less helpful features)
    - Biofeedback (positive features, less helpful features)
- Thinking back to treatment, at last interview you said xxx helped the most. What is your view now?
- Thinking back to treatment at last interview you said xxx concerned you. What is your view now.
- With time having passed, what is it you still remember most vividly from treatment?
- Have you had any other UI treatments? What made you go? How have those treatments been? (link to effect)

## Self Care/ self-exercise [focus on aspects of last 6 months]

- PFMT undertaken at home? :
  - Still doing exercise at home (where, when, how often)
  - what is easy? What is difficult?
- On-going with biofeedback (bought device?) If so explore use– where, when, how often, ease, difficulty
- Any other things you do to manage UI?
- Anything changed in way you exercise since we last spoke – what changed and why?
- Factors influencing adherence to home programme of exercise now that supervised treatment stopped. [possible probes below]
  - things that help you stick with exercise
  - things that stopped/hampered exercise
  - on-going routine? Any breaks? Manage to restart?
  - Other social influences (such as work commitments etc)
  - Other environmental influences (such as privacy at home etc)
  - Spoken with anyone else and has this changed what you do?
- How is your confidence to exercise now? Has it changed over time?
- Do you plan to keep going with exercise longterm? What would help you to do this or to restart if stopped?

## Links between intervention and outcome (if not been explicitly covered through previous content)

- Links between intervention and outcome: feeling now about whether or not treatment has made a difference. What are they; how do they make a difference; why do they make a difference?

## Closure

Thank you for talking with me today. The interview we have recorded will be removed from the recording device as soon as possible and stored securely. When the interview is typed up, all identifying information will be removed. We will study the information you have given us alongside that given by other women. We would like to speak with you again in a years time, that interview is usually undertaken by phone. Explain will write a month before and then call to find a good time; confirm consent to call.

**Exploring women’s experiences of symptoms and treatment: An interview study linked to the OPAL trial**

# 24 month Follow-up Interview (Interview D)

**RESEARCH QUESTION: To investigate women’s experiences of the interventions, both basic and intensive PFMT, to identify the barriers and facilitators which impact on adherence in the short- and long-term, to explain the process through which they influence adherence, and to identify whether these differ between randomised groups.**

## Re-introduction to self, interview, consent

Is this an OK time to call? Is there another time I can call back?

Thank you for agreeing to talk with me again. We greatly appreciate the help you are giving with the OPAL study. To recap the OPAL interview study is about women’s experience of UI and how they have got on with the treatment they have been given. I am xxx, one of the researchers on the OPAL study. Today’s interview will take approximately 15 minutes – is it still OK to record? Are you still happy to be interviewed? Do you have any questions for me before we start?

**In this interview we will focus on the last year (that is since we last spoke to you).**

## Ice breaker

How have you been generally since we last spoke?

## Symptoms [maintain focus on comparison to 12 months ago]

- Extent of UI symptoms now/ how bothered by them
- Comparison of symptoms now to 12 months ago (ie post intervention)
- Why do you think symptoms have changed/not changed over the last 12 months? [Focus on issues woman raised at 12 month interview and ask about intervention].

## Intervention

- Thinking back to treatment with therapist/nurse, what are your views on it now.
  - - PFMT (positive features, less helpful features)
    - Biofeedback (positive features, less helpful features)
- With time having passed, what is it you still remember most vividly from treatment?
- Have you had any other UI treatments? What made you go? How have those treatments been? (link to effect)
- Although considerable time has passed since you got the treatment, anything that has stuck with you? Anything you would change?

## Self Care/ self-exercise [focus on aspects of last 12 months]

- PFMT undertaken at home? :
  - Still doing exercise at home (where, when, how often)
  - what is easy? What is difficult?
- On-going with biofeedback (bought device?) If so explore use– where, when, how often, ease, difficulty
- Any other things you do to manage UI?
- Anything changed in way you exercise since we last spoke – what changed and why?
- Factors influencing adherence to home programme of exercise now that some time has passed since treatment. [possible probes below]
  - things that help you stick with exercise
  - things that stopped/hampered exercise
  - on-going routine? Any breaks? Manage to restart?
  - Other social influences (such as work commitments etc)
  - Other environmental influences (such as privacy at home etc)
  - Spoken with anyone else and has this changed what you do?
- How is your confidence to exercise now? Has it changed over last year?
- Do you plan to keep going with exercise longterm? What would help you to do this or to restart if stopped?

## Links between intervention and outcome (if not been explicitly covered through previous content)

- Links between intervention and outcome: feeling now about whether or not treatment has made a difference. What are they; how do they make a difference; why do they make a difference?

## Closure

Thank you for talking with me today. The interview we have recorded will be removed from the recording device as soon as possible and stored securely. When the interview is typed up, all identifying information will be removed. We will study the information you have given us alongside that given by other women.
